# Prognostic nutrition index as a predictor of coronary artery aneurysm in Kawasaki Disease

**DOI:** 10.1186/s12887-020-02111-y

**Published:** 2020-05-11

**Authors:** I-Hsin Tai, Pei-Lin Wu, Mindy Ming-Huey Guo, Jessica Lee, Chi-Hsiang Chu, Kai-Sheng Hsieh, Ho-Chang Kuo

**Affiliations:** 1 Kawasaki Disease Center and Pediatrics, Kaohsiung Chang Gung Memorial Hospital, Taiwan, College of Medicine, Chang Gung University, #123 Da-Pei Road, Niaosong District, Kaohsiung city, 83301 Taiwan; 2 Department of Pediatric Emergency China Medical University Children’s Hospital, China Medical University, Taichung City, Taiwan; 3 Department of Medicine, College of Medicine, China Medical University, Taichung City, Taiwan; 4 University of Maryland Medical Center, Baltimore, MD USA; 5 Department of Statistics, National Cheng Kung University, Tainan city, Taiwan; 6 Department of Pediatrics, Shuang Ho Hospital-Taiwan Medical University, New Taipei City, Taiwan

**Keywords:** Prognostic nutrition index, Kawasaki Disease, Coronary artery aneurysm, Albumin, Lymphocyte

## Abstract

**Background:**

Kawasaki Disease (KD) is considered a major acquired heart disease in children under the age of 5. Coronary artery aneurysm (CAA) can occur in serious cases despite extreme therapy efforts. Previous studies have reported low serum albumin level was associated with disease outcome, but no further investigation was addressed yet.

**Method:**

This retrospective (case-control) study randomly included children with KD who were admitted and underwent laboratory tests before undergoing IVIG treatment in this institution, the largest tertiary medical center in southern Taiwan from 2012 to 2016. Prognostic nutrition index (PNI), an albumin-based formula product, was evaluated as a predictor of CAA the first time. The progression of CAA was monitored using serial echocardiography for six months. We performed multivariable logistic regression analysis on the laboratory test and PNI with the disease outcome of the KD patients.

**Result:**

Of the 275 children, 149 had CAA, including transient dilatation, while the other 126 did not develop CAA during the 6-month follow-up period. A multivariate logistic regression model revealed that PNI, gender, IVIG non-responder, and platelet count are significant predictors of CAA with a 95% confidence interval estimator of 1.999, 3.058, 3.864 and 1.004, respectively. Using PNI to predict CAA presence gave an area under the receiver-operating-characteristics (ROC) curve of 0.596. For a cutoff of 0.5 in the logistic regression model and the PNI cut-off point is taken as 55 together with IVIG non-responder, boy gender, and platelet count take into account, sensitivity and specificity were 65.7 and 70.4%.

**Conclusion:**

PNI could be a candidate of adjunctive predictor of coronary artery aneurysm in addition to IVIG non-responder. Together with low PNI, IVIG non-responder, male gender and platelet count will give high odds to predict coronary artery aneurysm within 6 months of illness.

## Background

KD is the worldwide leading cause of acquired heart disease in developed countries, and the most serious sequela is the development of a CAA. Starting treatment with IVIG within 9 days of the onset of fever reduces the incidence of coronary artery aneurysms from 25% to 3 ~ 5% [1] in absolute luminal dimensions. The 2017 American Heart Association (AHA) scientific statement defined different management protocols for KD patients with and without regression of coronary artery aneurysm [2] 4–6 weeks after the onset of KD. This protocol difference demonstrates a delayed regression of coronary dilation, which indicates a more severe coronary vasculitis and deservedly more aggressive therapy and monitoring. Wu et al. showed that morbidity rates increased in those patients whose CAA regression occurred more than 2 months later [3]. Therefore, early or late regression of coronary vasculitis is crucial for future prognosis stratification.

PNI has been used to predict and evaluate post-operative status in cancer patients for decades, ever since it was first published in 1983 [4]. PNI has also been used to predict mortality in patients with ST-segment elevation myocardial infarction (STEMI) [5]. PNI is currently determined by albumin (ALB) and total lymphocyte count (TLC), while its original formula used triceps skinfold thickness (TSF), serum transferrin concentration (TFN), and delayed hypersensitivity reaction (DHC, no reaction = 0, < 5 mm induration =1, and > 5 mm induration = 2) instead of the current TLC. Albumin has been a consistent parameter in the PNI formula because various studies have shown its correlation with nutrition and immune status. By definition, a higher albumin level or lymphocyte count contributes to a greater PNI value, which indicates a superior self-healing ability due to sufficient nutrition and improved immune capacity, which can prevent opportunistic infectious pathogen invasion. In our previous report, we found that the serum level of albumin was associated with IVIG resistance in KD patients [6].

Although the definite cause of KD remains unknown, evidence [7] has shown that KD is most likely caused by an infectious agent(s) that produces a clinically apparent disease in genetically predisposed individuals. Once a patient develops KD, the vasculopathy cause plasma leakage as well as serum albumin. That explained the palmar and plantar erythema which usually accompanied by swelling in acute KD children. Hypoalbuminemia is wide known as risk factor for IVIG resistant KD, which correlated with CAA development. This study aimed to investigate whether there is causality between nutrition, an albumin-based status, and CAA in KD.

## Methods

### Subjects’ enrollment & data collection

This study was approved by Chang Gung Memorial Hospital’s institutional review board with IRB number 102-3595C. We performed a retrospective case control study with the clinical records of KD patients hospitalized at Kaohsiung Chang Gung Memorial Hospital between 2012 and 2016. We included patients diagnosed with KD based on AHA guidelines [2]. and collected data from KD with CAA (CAA present group) and age-matched KD population without CAA formation as the control group (CAA absent group). According to the latest AHA guideline, classic KD is diagnosed with the presence of fever for at least 5 consecutive days with at least 4 of the 5 principal clinical features (oral changes, conjunctivitis, cervical lymphadenopathy, extremity changes, and dysmorphism rash). If a patient has more than four of the principal clinical features together with limb induration, KD can be diagnosed with just 4 days of fever. All of the participating patients underwent 2D-echocardiography of the coronary artery during admission, as well as at 2, 4, and 6–8 weeks and 3, 4, and 6 months from disease onset. Positive echocardiogram findings of CAA were defined by a body surface area adjusted *Z* score of coronary segments exceeding 2.5 in accordance with AHA criteria [2]. All the patients were diagnosed with KD and underwent IVIG treatment in our hospital except three patients who were afebrile spontaneously within 5 days of illness. Patients who received IVIG treatment elsewhere were excluded. The following laboratory data were collected prior to administering IVIG: total white blood cell count (WBC), the percentage of neutrophils and lymphocytes, hemoglobin levels, platelet count (PLT), serum concentrations of C-reactive protein (CRP), aspartate aminotransferase (AST), alanine aminotransferase (ALT), and serum albumin.

Afterwards, PNI was calculated according to the serum level of albumin & total lymphocyte count [PNI = 10 x albumin (mg/dl) + 0.005 x lymphocyte counts (10^9^ L^− 1^)] as previously reported [4].

### Statistical analyses

All values are expressed as mean ± standard deviation (SD), median (1st quantile, 3rd quantile), or number (percentage), as appropriate. For all analytic results, a *p*-value of 0.05 is considered statistically significant. We adopted the independent t and Mann-Whitney *U* test to identify the difference between the two groups for continuous variables according to the normality test. For independent variables, Pearson chi-square test was applied to compare the proportion between both groups. We used the ROC curve to analyze the optimal cut-off point of a variable with Youden’s index criterion. To compare the odds ratio of significant variables, we selected the candidate variables using univariate logistic regression with a *p*-value of 0.05 and the final model using multivariate logistic regression. All statistical analysis was performed using SPSS statistical software for Windows version 13.0 (SPSS for Windows, version 13; SPSS, Chicago, IL).

## Results

We enrolled 275 patients with KD from our search database in this study. We randomly and retrospectively included 149 KD patients with CAA formation and 126 age-matched KD patients without CAA formation as the control group. The percentage of males was higher in the CAA present group (76.5% vs. 54.0%, *p* < 0.001) than the CAA absent group. We found no statistical differences in age for KD between the two groups (due to this being an age-matched case control study). The median age of these patients upon diagnosis of acute KD was 1.14 years and 1.31 years (*p* = 0.161), respectively (Table 1).

**Table 1:**
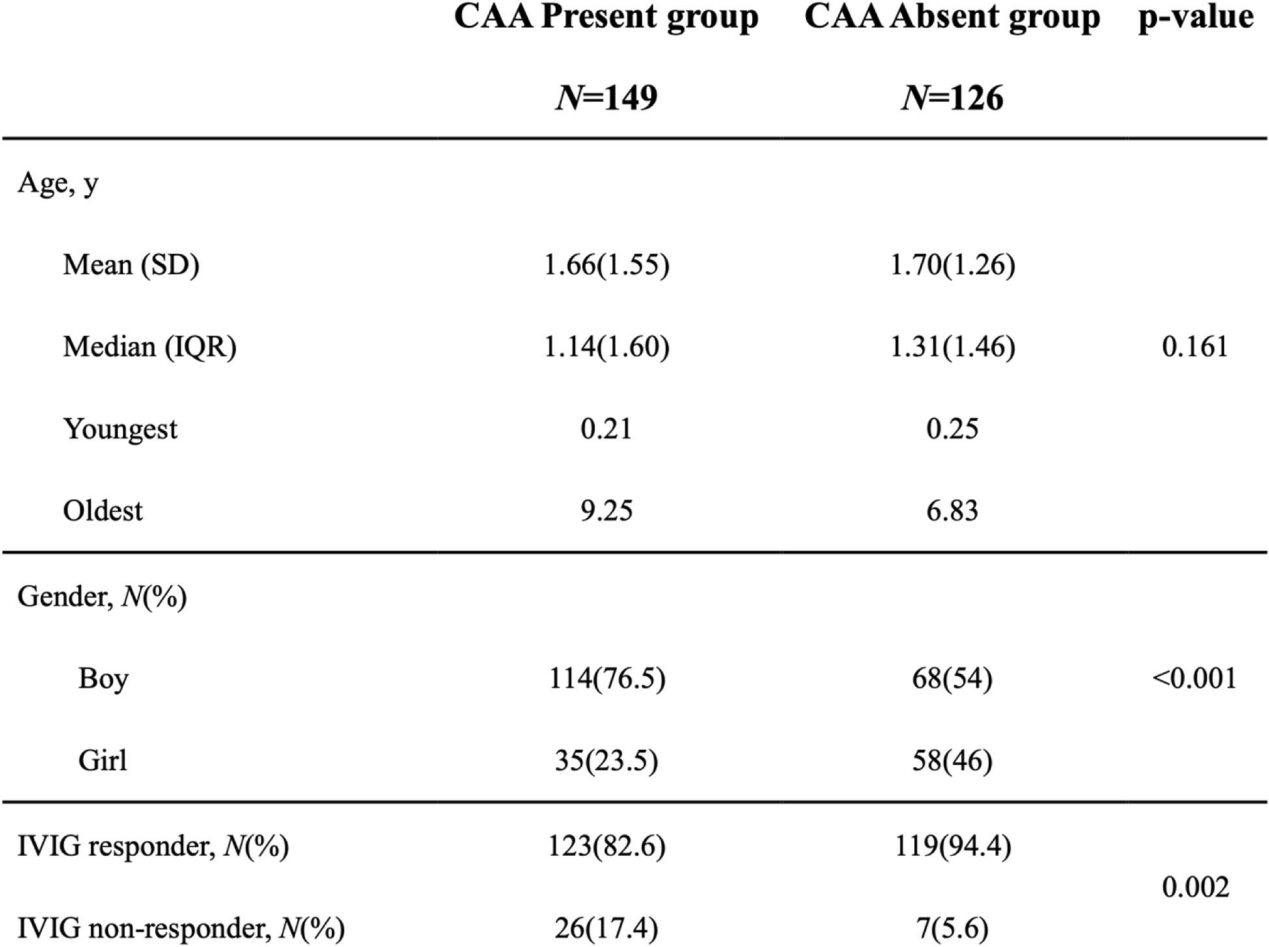
Characteristics of participants, *N=275*

The initial absolute values of the complete blood count, differential count, and CRP, as well as the liver function and albumin concentrations, in each group are provided in Table 2. We found WBC to be higher in the CAA present group than in the CAA absent group (13.5 vs. 12.7 × 10^3^/mm^3^, *p* = 0.050), the neutrophil count to be higher in the CAA present group (7.88 vs. 7.05 × 10^3^/mm^3^, *p* = 0.015) than in the CAA absent group, the platelet count to be higher in the CAA present group (360.0 vs. 314.5 × 10^3^/mm^3^), *p* = 0.001) than in the CAA absent group, the CRP levels to have no significant difference between the CAA present group (69.9 vs. 63.2 mg/L, *p* = 0.314) and the CAA absent group, and albumin levels to be significantly lower in the CAA present group (3.7 vs. 3.9 g/dL), *p* = 0.002) than the CAA absent group.

**Table 2:**
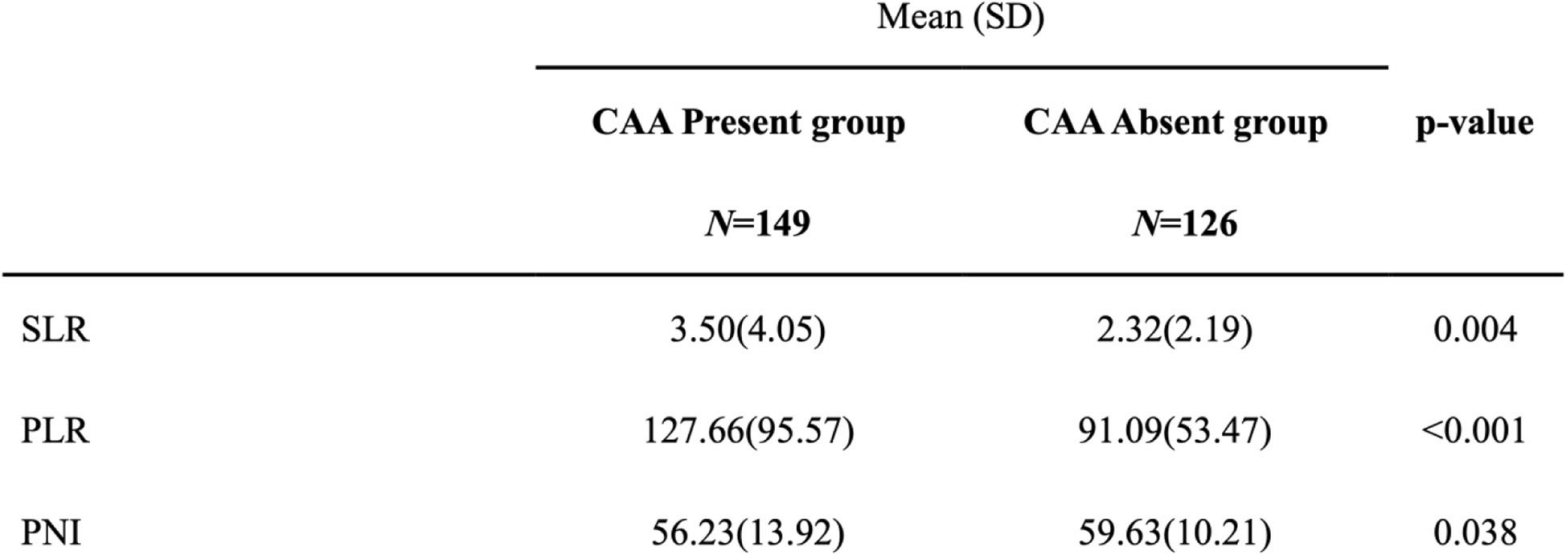
Baseline laboratory data of the CAA presence and absence group

Furthermore, we found that prior to IVIG therapy, the CAA present group had a significantly higher segment-to-lymphocyte ratio (SLR) (3.50 vs. 2.32, *P* = 0.004) and platelet-to-lymphocyte ratio (PLR) (127.66 vs. 91.09, *P* < 0.001), while a significantly lower PNI (56.23 vs. 59.63, *P* = 0.038), than the CAA absent group, as shown in Table 3.

**Table 3:**
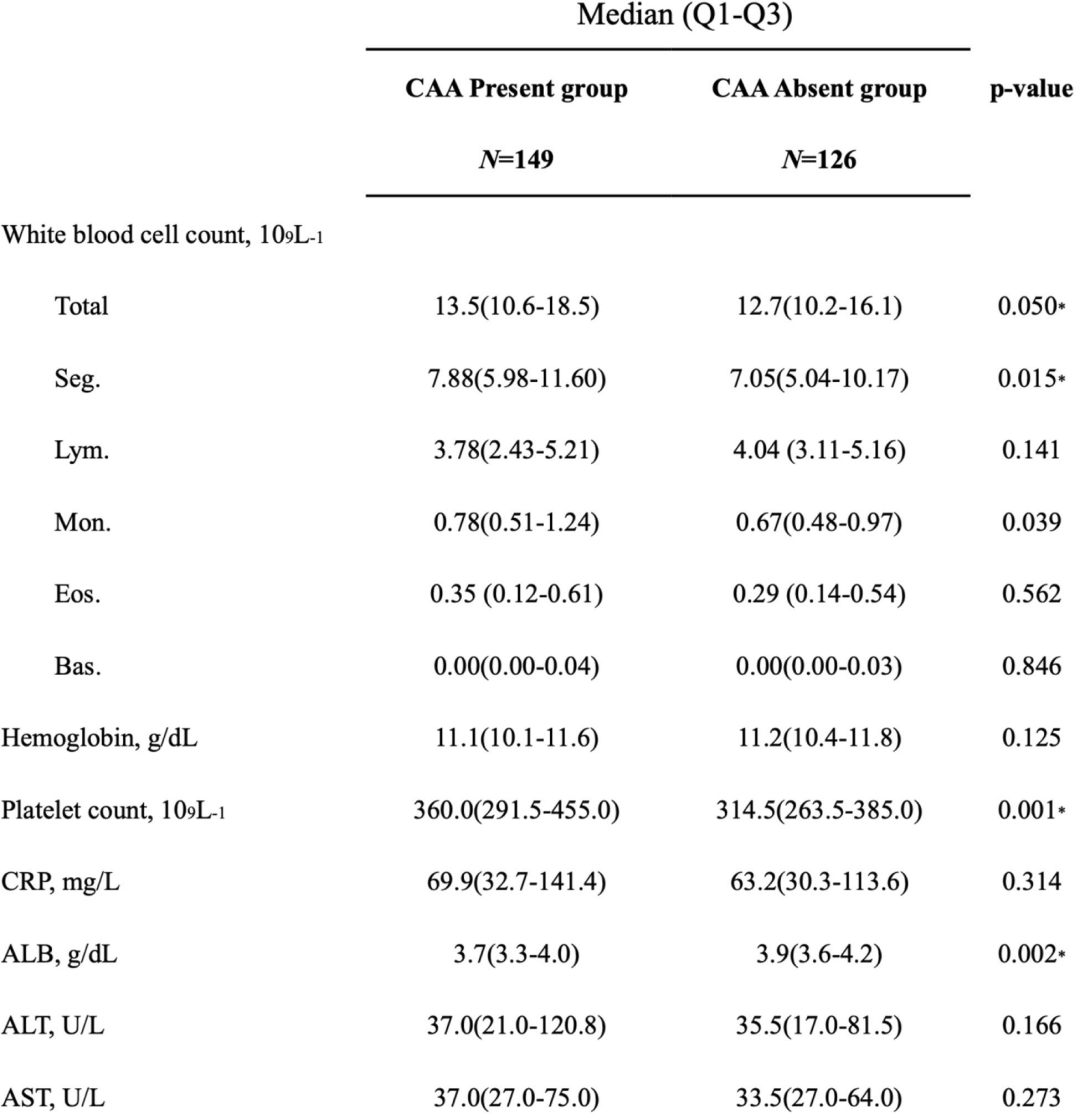
Blood cell ratio and PNI of CAA presence and absence group

**Fig. 1:**
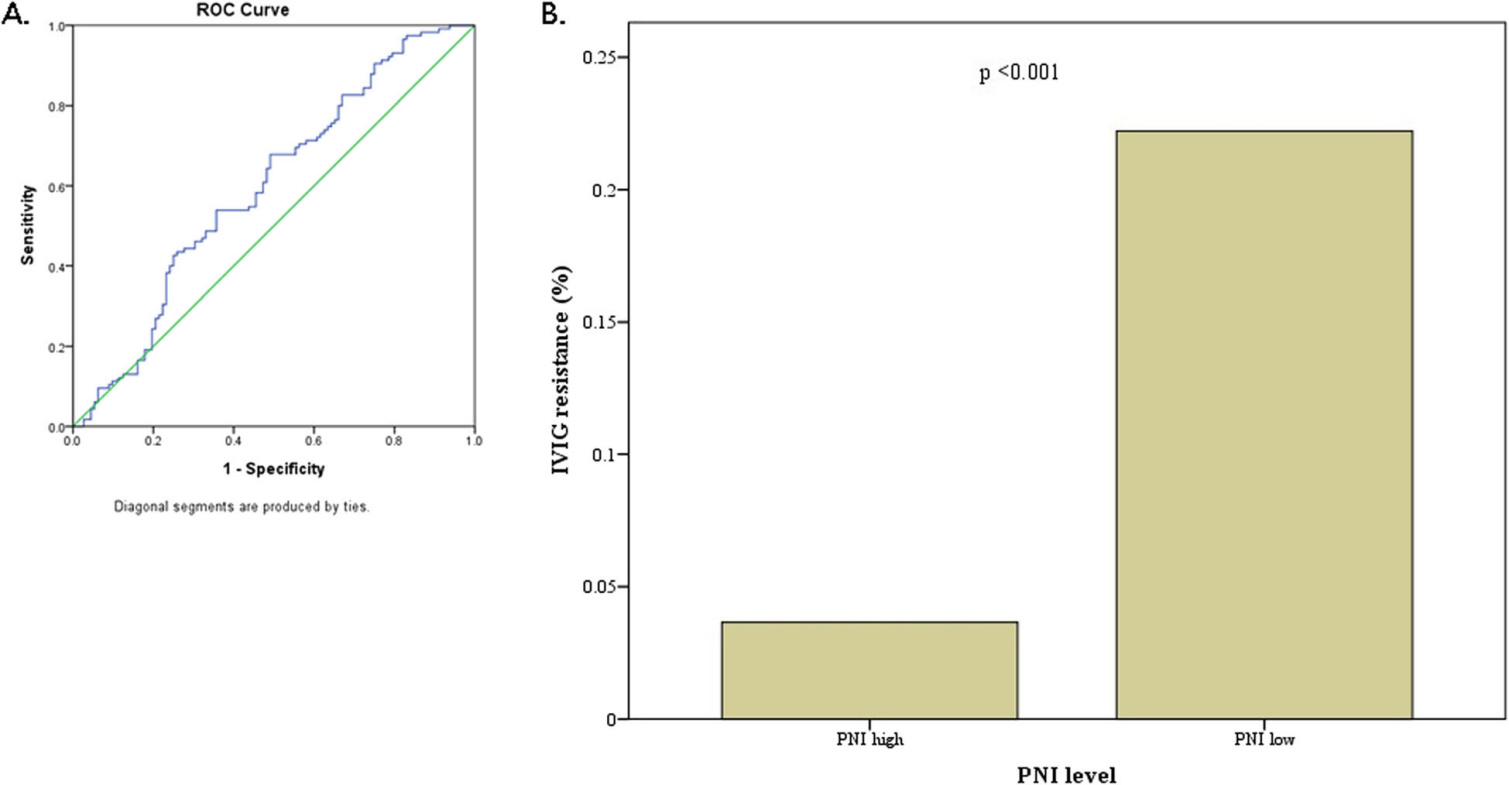
: PNI as predictor of CAL. A. The ROC curve analysis shows that the area under the ROC curve is 0.588 (0.513–0.663), with a significance of 0.023 for the prognostic nutritional index (PNI). The cut-off value of PNI is taken as 55.24, with a sensitivity of 0.500 and a specificity of 0.678 by maximizing the Youden’s index B. Low-PNI group has significant high odds (odds = 7.65) to be IVIG-resistant.

The ROC curve analysis (Fig. 1A) indicates that the area under the ROC curve is 0.588, with a significance 0.023 for PNI. The PNI cut-off value is determined to be 55.24 with a sensitivity of 0.500 and a specificity of 0.678 by maximizing the Youden’s index. In the following paragraph, we define the high-PNI group as PNI ≥ 55 and the low-PNI group as PNI < 55.

According to the multivariate analysis with logistic regression procedure (Table 4), the male gender, IVIG non-responder, elevated platelet counts, and PNI-low group positively correlated with the presence of CAA. The risk of CAA formation was 3.058 greater in boys and 3.864 greater in the IVIG non-responder. As for PNI-low group, the even earlier information acquired, the risk of CAA was nearly twice as PNI-high group. The odds of IVIG-resistant was 7.65 times greater for low-PNI patients than for high-PNI patients (Fig. 1B) (*p* < 0.001).

**Table 4:**
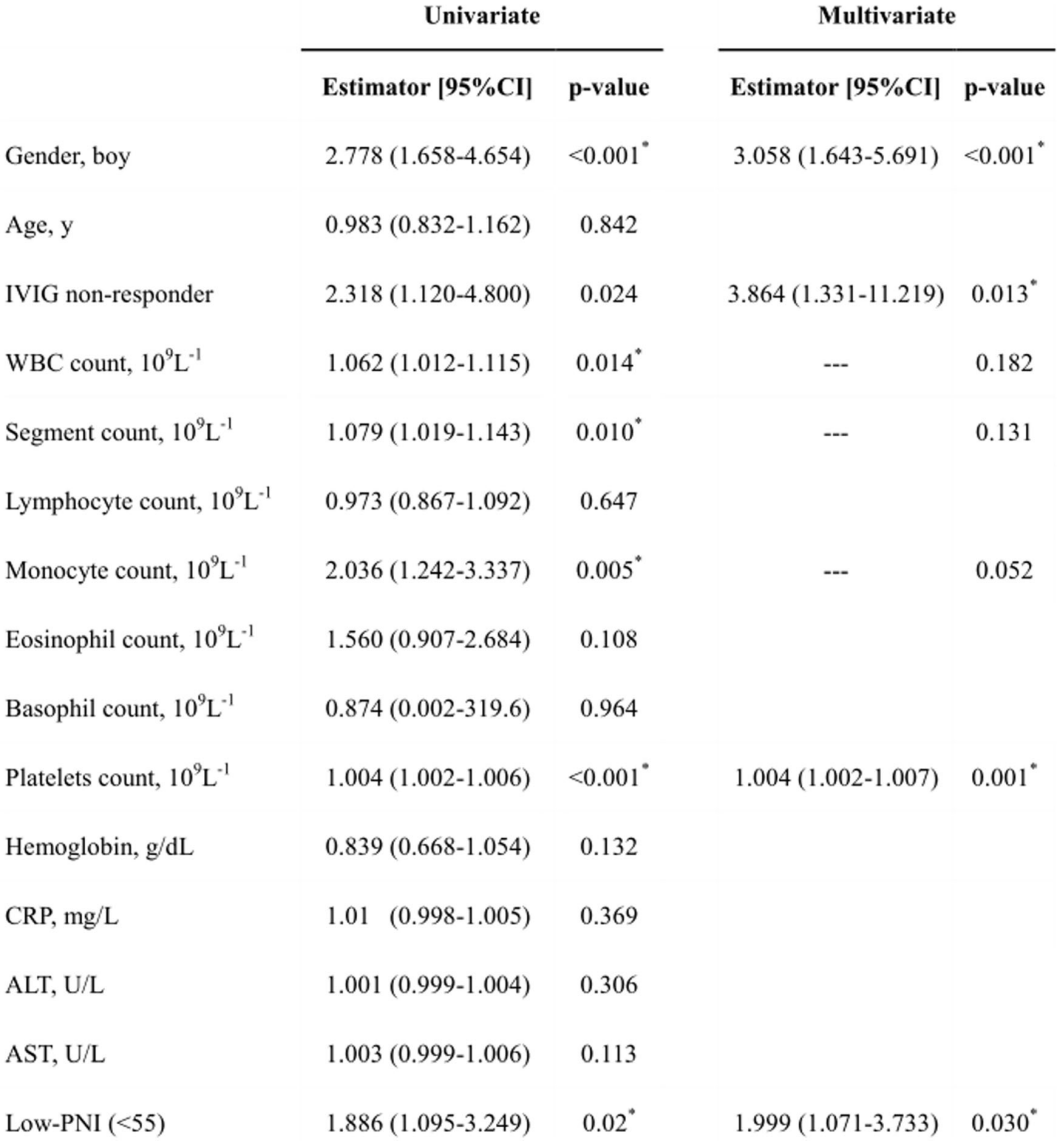
Univariate/multivariate logistic regression model with CAA group

Under multivariate analysis, male gender, higher platelet count and lower PNI value (< 55) before IVIG all had significantly positive correlation to CAA presence in 6 months of KD-illness.

## Discussion

### PNI role in the history

Nutrition assessment results have previously been proven to define the incidence of post-operative complications, mortality, and morbidity in patients with heart failure or malignant cancers [8–13]. While many nutritionists suggest using the Controlling Nutritional Status (CONUT) score to assess the nutrition status of acute heart failure, a large retrospective cohort study demonstrated that PNI has the same prognostic impact in patients with decompensated heart failure [14, 15]. PNI was an independent predictor for evaluating the correlation between nutritional status and malignancy or vital organ failure mortality by comparing subjects of the high-PNI and low-PNI groups [12, 16, 17]. In addition to being used with adult diseases, PNI can also predict the clinical outcome of the pediatric population in the intensive care unit after cardiac operation [18]. However, we found PNI could predict CAA risk in acute KD patients in addition to correlating with nutrition status.

#### Hypoalbuminemia in KD and CAL formation

KD is a form of chronic vasculitis that may last for months to years in regard to pathophysiology. Therefore, all KD patients with or without coronary ectasia are considered at high risk for accelerated atherosclerosis according to the epidemiological evidence and should undergo nutrition counseling and diet education in an effort to reduce their future cardiovascular burden [19]. Research has identified that younger than 6 months of age, male, incomplete KD, longer fever duration, higher CRP levels (> 100 mg/l), and lower albumin levels (< 35 g/L) were all independent risk factors for CAA formation [20], thus indicating that both delayed initiation of KD target therapy and hypoalbuminemia, which indicates a relatively poor nutritional status, result in higher incidence rates of CAA complications in patients with acute KD, despite the administration of IVIG therapy.

#### PNI predicts KD with CAA & IVIG non-responder

In the current study, we showed that PNI, an albumin based long-term predictor of cancer, was also a significant independent predictor of CAA in any coronary segment during the 6 months after the onset of illness (PNI < 55, estimator: 1.999, *p* = 0.030), as well as gender, IVIG non-responder, and platelet count. However, the associations of pre-treatment platelet count and CAA formation were relatively weak in this cohort, with a 95% confidence interval of estimator between 1.002–1.007. To the best of our knowledge, this study is the first to discuss the predictive value of PNI on CAA formation in KD patients before they receive initial IVIG therapy. Kobayashi et al. constructed a seven-variable predictive model to identify IVIG-resistant KD using pretreatment laboratory data. Although previous research has shown that most KD patients with CAA are unresponsive to IVIG, the detailed mechanism between IVIG non-responders and CAA formation has yet to be explained. Our results are in line with Kuo et al.’s previously published studies demonstrating the significant relationship between hypoalbuminemia and IVIG-resistant KD, which often indicates a higher incidence of CAA [6]. Of particular interest is the discrepancy conclusion from Japan [21] (Kobayashi et al., 2006) to Taiwan (Kuo et al., 2010) regarding the correlation between IVIG non-responder and hypoalbuminemia using multivariate logistic regression models [6, 21]. Assuming that both research methods were appropriately and strictly designed, we may presume that an unknown ongoing process involved nutrition status, in addition to vascular inflammation. However, early validation research on Japan scoring models yield inconsistent result between different races [2, 22–24]. It showed multiple ethnicity-exclusive models are required. Our findings revealed that a low pre-treatment PNI level (PNI < 55) correlated to a high incidence of CAA complication in KD patients, as well as IVIG non-responder.

#### PNI practice

Low-PNI alone before initial IVIG therapy have nearly 2-fold (estimator: 1.999, Table 4) risk to develop future CAA. In the setting of low-PNI, IVIG non-responder, male gender, and higher platelet count will give rise to at least 8.8-fold higher risk to develop CAA. Therefore, PNI in conjunction with IVIG response, gender, and platelet will have better prediction of developing CAA within 6 months of illness.

## Conclusion

The utility of PNI as adjunctive predictor of coronary artery aneurysm in addition to IVIG non-responder, male gender and platelet count will give high odds for predicting CAA formation in KD patients. The simply quick formula allow physicians to identify patients that may benefit from aggressive primary or advanced anti-inflammatory therapies.

## Data Availability

The datasets used and analyzed during the current study are available from the corresponding author on reasonable request.

## Abbreviations

KD: Kawasaki Disease
IVIG: intravenous immunoglobulin
CAA: coronary artery aneurysm
PNI: Prognostic nutrition index
